# Humanoid Robot Cooperative Motion Control Based on Optimal Parameterization

**DOI:** 10.3389/fnbot.2021.699820

**Published:** 2021-06-21

**Authors:** Qiubo Zhong, Yaoyun Li, Caiming Zheng, Tianyao Shen

**Affiliations:** ^1^Robotics Institute, Ningbo University of Technology, Ningbo, China; ^2^State Key Laboratory for Manufacturing Systems Engineering, Xi'an Jiaotong University, Xi'an, China

**Keywords:** humanoid robot, collaborative control, stability constraints, parameterized optimization control, energy optimization

## Abstract

The implementation of low-energy cooperative movements is one of the key technologies for the complex control of the movements of humanoid robots. A control method based on optimal parameters is adopted to optimize the energy consumption of the cooperative movements of two humanoid robots. A dynamic model that satisfies the cooperative movements is established, and the motion trajectory of two humanoid robots in the process of cooperative manipulation of objects is planned. By adopting the control method with optimal parameters, the parameters optimization of the energy consumption index function is performed and the stability judgment index of the robot in the movement process is satisfied. Finally, the effectiveness of the method is verified by simulations and experimentations.

## 1. Introduction

With the development of artificial intelligence and automation control technology, research, and the application of robots have become increasingly widespread. Current studies on robot control cooperation mainly focus on mobile multi-wheel robots (Jhang et al., 2019), multi-manipulator cooperation (Yu et al., 2019; Wang J. et al., 2020), and human-machine collaboration (Ikeda et al., 2021). The humanoid robot can generally be considered as a multi-coupled and non-linear dynamic system (Sanprasit, 2020), which increases the complexity of the collaboration between humanoid robots. However, due to its flexible and firm multi-joint structure, the idea of using humanoid robot technology to complete sophisticated and tedious tasks has gradually matured. In particular, humanoid robots have gradually become an ideal carrier for production operations and transportation. However, when the weight of the heavy object carried is too large, a single humanoid robot carries a heavy object will fail to complete the work successfully and even may fall down and damage the joints. And in the fields such as rescue tasks (Hong et al., 2018), logistics handling cooperation (Straßmann et al., 2019), and sports competition (Wang Z. et al., 2020), broad applications are expected on humanoid robots. Therefore, the problem of multi-humanoid robot cooperation has gradually become a hot topic in the field of robotics.

In the process of cooperative movement of two humanoid robots, unbalanced external forces and inconsistent speeds are among the main factors causing unexpected consequences such as falling and damage (Wu et al., 2016; Rojas et al., 2021). To avoid such a situation, each robot must maintain a harmonious and stable gait as much as possible. The process of co-operation of humanoid robots carrying heavy objects is a nonlinear control problem with a high degree of freedom and complexity (Wu et al., 2016; Rioux et al., 2017). The analysis of stability in the cooperative process is the prerequisite for the movement control. Researchers introduced the linear inverted pendulum model and the principle of Zero-Moment Point (ZMP) dynamic balance (Sugihara et al., 2002; Kajita et al., 2003), in order to achieve the stable walking of the humanoid robot via regulating the angle of the joints. However, as the mass of legs in the robot has been ignored, computational errors are inevitable in the method mentioned above. Kashyap and Parhi (2021) established a hybrid controller based on a linear inverted pendulum coupled with a flywheel to produce a robust expected walking gait in the experiment, but analysis and calculation are more complicated. Wu et al. (2016) proposed a symmetrical hybrid control framework work to ensure the synchronization of movements among multiple robots based on the interaction between the leader and the follower. However, this method requires that consideration be given to the velocity between the two robots. Yang et al. (2019) convert the problem of cooperative handling of humanoid robots into a quadratic planning problem to determine the trajectory of the joints, but the inequality constraints will lead to higher computational costs. Keerio et al. (2008) used teleoperation among two humanoid robots to correct the position offset in real time.

In previous studies of robotic stability problems based on the ZMP method, energy consumption during robot motion is generally undiscussed. The robot's low energy consumption can effectively reduce the output torque of the articulated motor and protect the robot (Qiang et al., 2015). Liu et al. (2021) used a direct collocation method that converts the trajectory optimization problem into a nonlinear programming problem which can reduce energy consumption and improve the stability in the multi-phase gait motion process, but usually the local optimal solution is found. Ding et al. (2018) took the acceleration of the center of mass as the evaluation criteria of energy consumption to verify the energy-saving performance in the stable zone of the ZMP. However, for multi-joint robots, this method usually takes more time-consuming iteration to calculate joint torques and velocities in advance. Wang et al. (2011) proposed energy consumption estimation strategies and energy efficiency optimization algorithms based on important energy consumption indicators (average power, average power deviation, and average torque loss), fulfilling low-energy gait based on ZMP stability criterion. However, the effect of the yaw moment on the stability was not taken into consideration. Thus, as the walking time increases, it will gradually lose stability. Shin and Kim (2014) minimized the energy consumption of the leg joints based on the three-mass inverted pendulum model and verified the energy-saving performance of each ZMP area. However, in order to compensate for the larger modeling error with greater mass and inertia, the maximum available AZR may be more limited.

In order to achieve stable walking and low energy consumption in cooperative tasks, it is necessary to establish a dynamic model that conforms to the humanoid robot cooperation. Meanwhile, the torque of each joint is expected to be measured for the computation of energy consumption of each joint using the dynamic model. Zhang et al. (2005) utilized the incomplete constraint characteristics of the differential gear train to achieve the goal of human-machine cooperation. Lagrange equation and D-H coordinate transformation method were adopted in the dynamic analysis of modular collaborative robots (Zhang et al., 2005; Ma et al., 2017). For flexible robots, Yin (2018) further studied the dynamic control based on rigid-flexible coupling dynamics. Pan et al. (2019) established a dynamic model of the collaborative robot based on the Newton-Euler method, analyzing the inertial parameters and joint friction parameters, as well as calculating the dynamic torque of the joint. And this method independently identifies the inertial parameters of each connecting rod assembly with the high precision of parameter identification. Hawley and Suleiman (2019) proposed a complex integrated control framework consisting of a centralized controller, an arm controller, and a ZMP preview controller. However, this method simplifies the coupling force in the collaboration to an external force, making it difficult to accurately measure the essential physical parameters during the motion process.

To address optimal control problems with continuous-time nonlinear constraints, parameterized control is an effective method. Zeng (2018) made the use of parametric control (Jennings et al., 1991) to solve the path planning problem of free-floating robots via transforming the original optimal control problem into an approximate optimal parameter selection problem. Wu et al. (2019) applied constraint conversion and smoothing function to convert the optimal PID parameter tuning problem of the flexible joint manipulator into a continuous state inequality constraint optimal parameter selection problem (Jennings et al., 1991), and subsequently realized the optimization of the flexible joint manipulator control.

Quadratic programming (QP) controller has been widely used in humanoid control in recent years. The QP controller can solve the constraints and objectives of the optimization problem in real time. Yang et al. (2019) solved the quadratic programming problem with inequality or equality constraints to determine the joint trajectory of the whole body. Zhang et al. (2004) transferred both velocity-level and acceleration-level redundancy-resolution schemes into a quadratic programming problem with equality and inequality constraints to solve joint torque optimization of redundant manipulators subject to physical constraints. Yang et al. (2018) solved QP problem on-online to optimize joint torques for massive object transportation by a humanoid robot. The controller, which is based on the QP of the inverse kinematics module, proposed by Feng et al. (2015) is an extension to the damped least squares method, and successfully implemented on Altas with a more reliable and safer performance. Otani et al. (2018) used a multi-robot QP (MRQP) controller to explicitly model the whole-body dynamics of both the human and the robot, which is not only keep balanced of robot, but also assist human in achieving common objectives. However, the QP controller cannot make the energy consumption globally optimal, and it is easy to cause joint mutation and affect the stability of the robot. The parameterized control method can address this problem well. QP controller has been widely used. Hence, the MRQP is considered as normal control in this paper, and the parameterized control method proposed in this paper is compared with it.

This paper investigates the optimal control of the humanoid robot cooperative manipulation issue. The idea of parameterized optimal control is introduced, which can resolve the two defects of instability caused by joint mutations and higher energy by comparing with the QP controller method. And the energy consumption of the robot in collaboration is adopted as an index function to optimize the parameters. Through the method of time scale transformation and constraint transformation, the continuous time state constraint is transformed into the optimal parameter selection problem, and the ZMP is found to be satisfied. The stability basis and the solution of the constraint conditions ensure the stability of the entire collaborative robot system while minimize the energy consumption of humanoid robots for collaborative handling.

## 2. Collaborative Robot Model Construction and Problem Description

**Figure 1:**
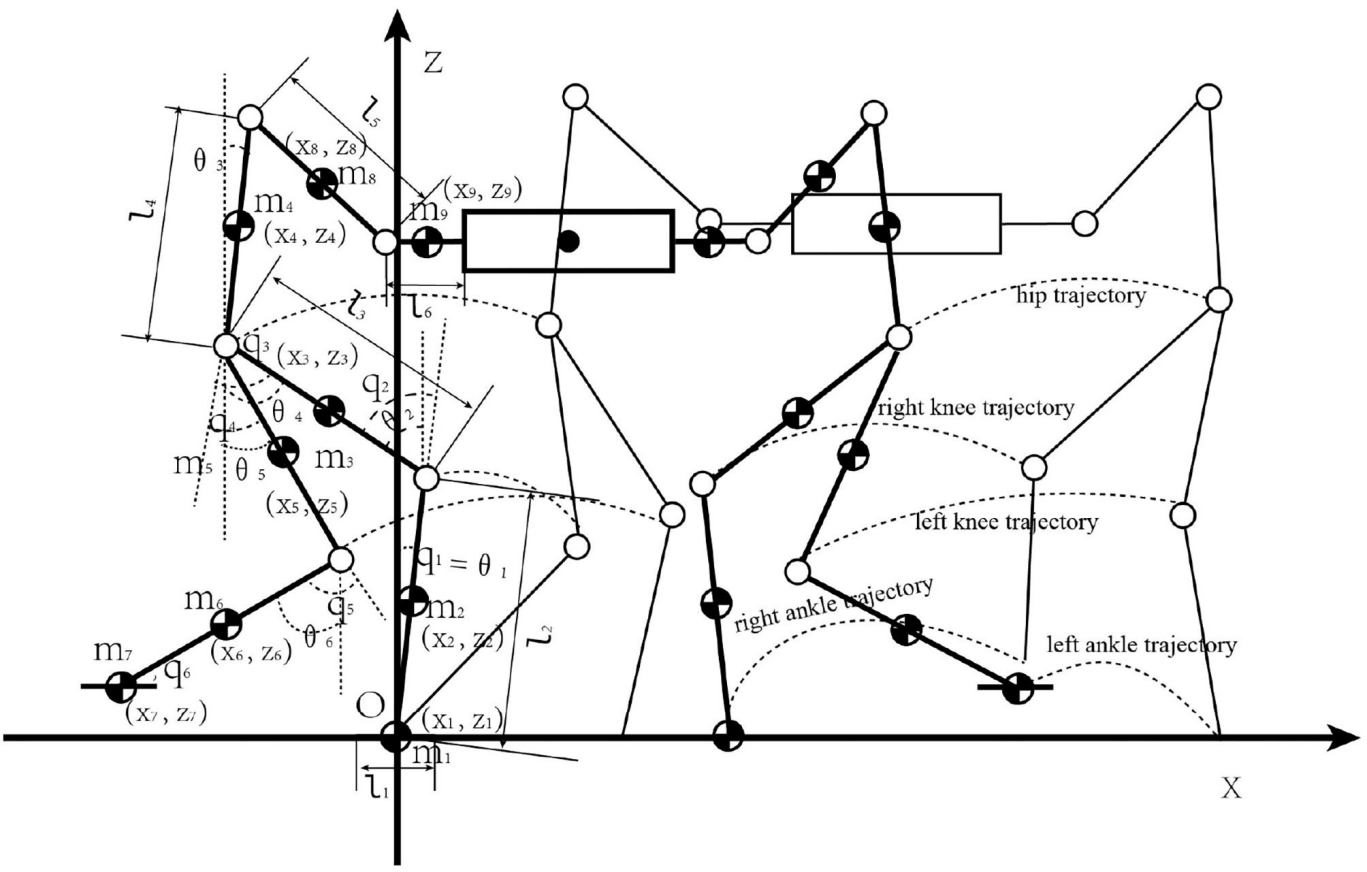
Humanoid robots cooperatively carry a heavy object model diagram.

This paper studies a complex dynamic walking system composed of two cooperative humanoid robots and a heavy object. A 9-link model with 6 degrees of freedom was established. The motion model on the vertical plane (x-z plane) is shown in Figure 1. Assuming that the mass of each link is concentrated at the center of mass, denoted by *m*_*i*_, the center of mass coordinates can be expressed as (*x*_*i*_,*z*_*i*_), where i = 1,2,…,9. The foot length link, calf link, thigh link, torso link, big arm link and forearm link are *l*_1_, *l*_2_, *l*_3_, *l*_4_, *l*_5_, *l*_6_. θ_*i*_, [i = (1,2,…,6)] is the angle between the connecting rod and the vertical line of the ground. A six-dimensional vector is used to represent the rotation angle vector of each joint of the connected robot j, that is, *****q***** = [*q*_1_, *q*_2_, *q*_3_, *q*_4_, *q*_5_, *q*_6_]^*T*^, which represents the forward rotation angles of the ankle, knee, and hip joints of the left and right legs, respectively. From the geometric relationship, it is written:

$$\begin{array}{l} q=K\theta +\delta \\ K=\left[\begin{array}{cccccc} 1 & 0 & 0 & 0 & 0 & 0\\ 1 & 1 & 0 & 0 & 0 & 0\\ 0 & 0 & 1 & 1 & 0 & 0\\ 0 & 0 & 1 & 0 & 1 & 0\\ 0 & 0 & 0 & 0 & 1 & 1\\ 0 & 0 & 0 & 0 & 0 & -1 \end{array}\right] \end{array}$$

where **δ** = [0 0 0 0 0 90]^*T*^.

The cooperative humanoid robot is described by the following dynamic equation:

$$\begin{array}{l} A\left(q\right)\ddot{q}+B\left(q,\dot{q}\right)\dot{q}+C\left(q\right)=\lambda \\ {\lambda =\lambda }_{r}+\lambda_{e} \end{array}$$

where **λ** is the total torque, *****q***** is the angle between the joint and the perpendicular to the ground, $\dot{q}$ and $\ddot{q}$ are the angular velocity of the joint rotation and Angular acceleration, respectively, *****A***(***q***)** is the inertial matrix, $B\left(q,\dot{q}\right)$ is composed of centripetal force and coriolis force, which is a first-order differential matrix, *****C***(***q***)** is a matrix related to gravity, **λ**_*e*_ is a cooperative coupling moment, **λ**_*r*_ is the control torque acting on the joint.

Selecting state variable as $x={\left[q^{T},{\dot{q}}^{T}\right]}^{T}$, the dynamic equation can be described in the form of the following state equation:

$$\begin{array}{l} \;\dot{x}\left(t\right)=f\left(t,x,u\right)\\ f\left(t,x,u\right)=\left[\begin{array}{cc} 0 & E_{p}\\ 0 & -A^{-1}\left(q\right)B\left(q,\dot{q}\right) \end{array}\right]x+\left[\begin{array}{c} 0\\ A^{-1}\left(q\right) \end{array}\right]u\\ \;+\left[\begin{array}{c} 0\\ -A^{-1}\left(q\right) \end{array}\right]C\left(q\right) \end{array}$$

where *t* represents a certain moment, *****u***** represents the control variables, *****E*****_*p*_ represents the unit matrix of the size of *p* × *p*.

This paper uses energy consumption as the optimal control index function:

$$\begin{array}{l} L_{0}\left(t,x\left(t\right),u\left(t\right)\right)=\sum_{i}m_{i}|{\dot{\xi }}_{i}\left(t\right){\ddot{\xi }}_{i}\left(t\right)|\\ E\left(t_{f}\right)=\int_{0}^{t_{f}}L_{0}\left(t,x\left(t\right),u\left(t\right)\right)dt \end{array}$$

where *m*_*i*_ indicates the mass of the *i*-th connecting rod, ${\dot{\xi }}_{i}\left(t\right)$ and ${\ddot{\xi }}_{i}\left(t\right)$ indicate the velocity and acceleration of the centroid of link *i* at the moment *t*, *L*_0_(*t*, *****x*****(*t*), *****u*****(*t*)) indicates the energy consumption index at time *t*, *t*_*f*_ indicates the terminal time, *E*(*t*_*f*_) represents the energy consumption function.

## 3. Continuous State Constraints

The cooperative motion process of humanoid robots is a continuous motion state, as the conditional constraints must be satisfied during continuous time. To ensure that the stability of the robot meets the constraints of the extended ZMP, the ZMP is required to remain within the bottom support polygonal area. Under the actual physical application conditions, it is necessary to meet the joint rotation angle constraint and the joint motor rated torque. The optimal control strategy must comply to the joint angular velocity constraints and avoid the rapid reaction of the joints effecting the stability.

### 3.1. Extended ZMP Constraint

In the process of cooperation of humanoid robots, ZMP can ensure that the robot presents a stable state of motion within the support polygon area. ZMP can be expressed by (*p*_*x*_, *p*_*y*_):

$$\begin{array}{l} p_{x}=\frac{\sum_{i=1}^{N}\left(m_{i}\left({\ddot{z}}_{i}+g\right)x_{i}-m_{i}{ẍ}_{i}z_{i}-\Theta_{iy}{\ddot{\Gamma }}_{iy}\right)}{\sum_{i=1}^{N}m_{i}\left({\ddot{z}}_{i}+g\right)}\\ p_{y}=\frac{\sum_{i=1}^{N}\left(m_{i}\left({\ddot{z}}_{i}+g\right)y_{i}-m_{i}{ý}_{i}z_{i}-\Theta_{ix}{\ddot{\Gamma }}_{ix}\right)}{\sum_{i=1}^{N}m_{i}\left({\ddot{z}}_{i}+g\right)} \end{array}$$

where *m*_*i*_ is the mass of the *i*-th link, *i* = 1, 2, …, *N, N* is the number of links, (*x*_*i*_, *y*_*i*_, *z*_*i*_) is the center of mass coordinates of the *i*-th connecting rod, ${ẍ}_{i},{\ddot{z}}_{i}$ represent the acceleration in the *x* and *z* directions respectively, *g* is the acceleration of gravity, and Θ_*ix*_ represents the moment of inertia of the *i*-th connecting rod around the *x*-axis, ${\dot{\Gamma }}_{ix}$ is the absolute acceleration component of the *i*-th connecting rod's center of mass.

The ZMP trajectory should be kept within the bipedal support area and meet the following conditions:

$$\begin{array}{l} SUP_{x\text{\_}min}\left(t\right)\leq p_{x}\leq SUP_{x\text{\_}max}\left(t\right)\\ SUP_{y\text{\_}min}\left(t\right)\leq p_{y}\leq SUP_{y\text{\_}max}\left(t\right) \end{array}$$

where [*SUP*_*x*_*min*_(*t*), *SUP*_*x*_*max*_(*t*)] and [*SUP*_*y*_*min*_(*t*), *SUP*_*y*_*max*_(*t*)] indicate the feasible range of the supporting polygon in the *x* and *y* directions at time *t*, respectively, expressed in *SUP*_*area*_ on a two-dimensional plane, that is, for ∀*t*, always satisfies (*p*_*x*_, *p*_*y*_) ∈ *SUP*_*area*_.

### 3.2. Joint Angle Constraint

Considering that the range of the rotation angle of each joint of the humanoid robot is limited, and each joint is restricted within the normal rotation range, the following state constraints are introduced:

$$\begin{array}{l} q_{i\text{\_}min}\leq q_{i}\left(t\right)\leq q_{i\text{\_}max},t\in \left[0,T\right] \end{array}$$

where *q*_*i*_(*t*) represents the angle of the *i*-th joint at time t, *q*_*i*_*min*_ represents the minimum joint rotation angle of the *i*-th joint under normal action, and *q*_*i*_*max*_ represents the maximum joint rotation angle of the *i*-th joint under normal action value.

### 3.3. Angular Velocity Constraint

Considering that the humanoid robot moves as smoothly as possible during the control process, limitation is applied to the angular velocity of the joints as the following state constraints:

$$\begin{array}{l} {\dot{q}}_{i\text{\_}min}\leq {\dot{q}}_{i}\left(t\right)\leq {\dot{q}}_{i\text{\_}max},t\in \left[0,T\right] \end{array}$$

where ${\dot{q}}_{i}\left(t\right)$ represents the angular velocity of the *i*-th joint at time *t*, ${\dot{q}}_{i\text{\_}min}$ represents the minimum rotational angular velocity of the *i*-th joint, and ${\dot{q}}_{i\text{\_}max}$ represents the maximum value of the rotational angular velocity of the *i*-th joint.

### 3.4. Control Parameter Constraint

Considering the rated torque of the joint motor of the humanoid robot, the following constraints are introduced:

$$\begin{array}{l} \omega \geq |u\left(t\right)|,t\in \left[0,T\right] \end{array}$$

where **ω** is the rated torque of the joint motor.

Therefore, the optimal control problem of the two cooperative humanoid robots can be expressed by the problem P, that is,

$$\begin{array}{l} minE\left(u,q\right)=E\\ s.t.\;\dot{x}\left(t\right)=f\left(t,x,u\right)\\ SUP_{x\text{\_}min}\left(t\right)\leq p_{x}\leq SUP_{x\text{\_}max}\left(t\right)\\ SUP_{y\text{\_}min}\left(t\right)\leq p_{y}\leq SUP_{y\text{\_}max}\left(t\right)\\ q_{i\text{\_}min}\leq q_{i}\left(t\right)\leq q_{i\text{\_}max},t\in \left[0,T\right]\\ {\dot{q}}_{i\text{\_}min}\leq {\dot{q}}_{i}\left(t\right)\leq {\dot{q}}_{i\text{\_}max},t\in \left[0,T\right]\\ \omega \geq |u\left(t\right)|,t\in \left[0,T\right] \end{array}$$

## 4. Parameterized Optimal Controller Design

Problem P is an optimal control problem with continuous state inequality constraints. This type of problem has continuous state inequality constraints and it is very difficult to be solved by traditional methods. It is required to satisfy the constraint conditions in the entire time period, which is equivalent to an infinite number of constraints. Based on the parametric control idea (Jennings et al., 1991), this paper transforms an infinite-dimensional optimal control problem into a finite-dimensional optimal parameter selection problem through the constraint conversion method together with the idea of local smoothing.

### 4.1. Control Parameterization

Considering the new time variables s ∈ [0,1], the time scale transformation is defined, which turns t ∈ [0,T] into s ∈ [0,1] as:

$$\begin{array}{l} \frac{dt}{ds}=tan\beta =T \end{array}$$

The initial condition is *t*(0) = 0, and the terminal condition is *t*(1) = *t*_*f*_, where *t*_*f*_ is the terminal condition.

A set of monotonically increasing sequence, *s*_*i*−1_ < *s*_*i*_, *i* = 1, 2, …, *n*_*p*_ satisfies *s*_0_ = 0, *s*_*n*_*p*__ = 1 and a set of parameters γ_*i*_, *i* = 1, 2, …, *n*_*p*_, constructing the following function:

$$\begin{array}{l} v\left(s\right)=\sum_{i=1}^{n_{p}}\gamma_{i}\chi_{\left[s_{i-1},s_{i}\right)}\left(s\right) \end{array}$$

Where the function *v*(*s*) is a piecewise constant control function, γ_*i*_ > 0, *i* = 1, 2, …, *n*_*p*_ are decision variables, and χ_[_*s*__*i*−1_, *s*_*i*_)_(*s*) is a symbolic function, which can be denoted:

$$\chi_{\left[s_{i-1},s_{i}\right)}(s)=\{\begin{array}{ll} 1, & s\in [s_{i-1},s_{i})\\ 0, & otherwise \end{array}$$

### 4.2. Constraints Transformation

The continuous state inequality constraints are independent of the control system. The inequality constraints must be satisfied in continuous time, which is equivalent to countless state inequality constraints in this time period. Using the method of constraint transformation, a series of continuous state inequality constraints are transformed into parameter optimization problems. For the constraint condition, *g*_*j*_(*s*, *****x*****(*s*)) ≥ 0, ∀*s* ∈ [0, 1] is equivalent to:

$$\begin{array}{l} L\left(x\left(s\right)\right)=\int_{0}^{1}min\left\{g_{j}\left(s,x\left(s\right)\right),0\right\}ds=0,j=1,2,...,n_{c} \end{array}$$

where *n*_*c*_ represents the number of constraints. The integral *L*(*x*(*s*)) is a non-smooth function, and it is undifferentiable. The smoothing algorithm is introduced to approximate the smooth function *L*_*j*, ϵ_(*g*_*j*_) to the original function *min*{*g*_*j*_, 0} as:

$$L_{j,\epsilon }(g_{j})=\{\begin{array}{cc} g_{j}, & g_{j}\leq -\epsilon \\ -\frac{{\left(g_{j}-\epsilon \right)}^{2}}{4\epsilon }, & -\epsilon \leq g_{j}\leq \epsilon \\ 0, & g_{j}\geq \epsilon \end{array}$$

where ϵ > 0 is usually a very small value, and *L*_*j*, ϵ_(*g*_*j*_) is a differentiable function. Let

$$\begin{array}{l} G_{j,\epsilon }\left(x\left(s\right)\right)=\int_{0}^{1}L_{j,\epsilon }\left(g_{j}\left(s,x\left(s\right)\right)\right)ds \end{array}$$

when ι is small enough, ∃ι(ϵ) > 0, and satisfies 0 < ι < ι(ϵ). For ∀ι can make *G*_*j*, ϵ_(*****x*****(*s*)) − ι ≥ 0 similar to *L*(*****x*****(*s*)).

For the joint angle constraint:

$$\begin{array}{l} g_{1}\left(t\right)=q_{i\text{\_}max}-q_{i}\left(t\right)\geq 0,g_{2}\left(t\right)=q_{i}\left(t\right)-q_{i\text{\_}min}\geq 0 \end{array}$$

For the joint angular velocity constraint:

$$\begin{array}{l} g_{3}\left(t\right)={\dot{q}}_{i\text{\_}max}-{\dot{q}}_{i}\left(t\right)\geq 0,g_{4}\left(t\right)={\dot{q}}_{i}\left(t\right)-{\dot{q}}_{i\text{\_}min}\geq 0 \end{array}$$

For the joint control torque constraint:

$$\begin{array}{l} z_{1}\left(t\right)=\omega -|u\left(t\right)|\geq 0 \end{array}$$

Problem P is expressed as:

$$\begin{array}{l} minE\left(u,q\right)=E\\ s.t.\;\dot{x\left(t\right)}=f\left(t,x,u\right)\\ \left(p_{x},p_{y}\right)\in SUP_{area}\\ g_{i}-\iota \geq 0,i=1,2,...,n_{c}\\ z_{j}-\iota \geq 0,j=1,2,...,n_{p} \end{array}$$

where *n*_*c*_ is the number of constraint conditions, and *n*_*p*_ is the number of parameter constraints of the control system.

Algorithm flow for solving problem P as shown in Table 1.

**Table 1:** Algorithm for solving problem P.

**Algorithm 1: Solve problem P**
Initial: $\epsilon =10^{-2},\iota =\epsilon /8,\epsilon_{m}in=10^{-4},u=u_{0}$ and *E* = *E*_0_
Input:*u*_0_, *E*_0_ and *n*_*p*_
Output: $u_{\epsilon ,\iota }^{*},E_{\epsilon ,\iota }^{*}$
(1): Solve problem P with *u, E* as initial point and *outputu*_ϵ, ι_*andE*_ϵ, ι_
(2): IF *g*_*j*_(*t*) − ι ≥ 0, ∀*t* = [0, *T*], *j* = 1, 2, …, *n*_*c*_ THEN goto (6)
(3): ElSE goto (5)
(4): SET ϵ = ϵ/10, ι = ι/10, *u* = *u*_ϵ, ι_, *E* = *E*_ϵ, ι_ and goto (1)
(5): SET ι = ι/2 and goto (1)
(6): IF ι ≥ ι_*min*_,THEN goto (1).
(7): ELSE stop and output $u_{\epsilon ,\iota }^{*},E_{\epsilon ,\iota }^{*}.$

## 5. Experiments

In order to verify the effectiveness of the method proposed in this study, numerical simulations of ZMP stability and optimal control were conducted in MATLAB. The experimental verification was carried out on the 6th generation of NAO robot, whose weight is about 5.48 kg^1^. In this paper, the optimal control computational software MISER 3.2 (Jennings et al., 1991) is used to solve the problem. And the proposed parameterized control method is compared with multi-robot QP (MRQP) (Otani et al., 2018) which is regarded as normal control. At the same time, in order for the two robots to be stable and safe to achieve the optimal goal during the process of carrying a heavy object, the angle parameters of the robot should meet the following range, taking the left leg of the robot in forward walking as an example: *q*_1_ ∈ (−68.15, 52.86), *q*_2_ ∈ (−5.29, 121.04), and *q*_3_ ∈ (−88.00, 27.73).

### 5.1. Stability Verification Analysis

**Figure 2:**
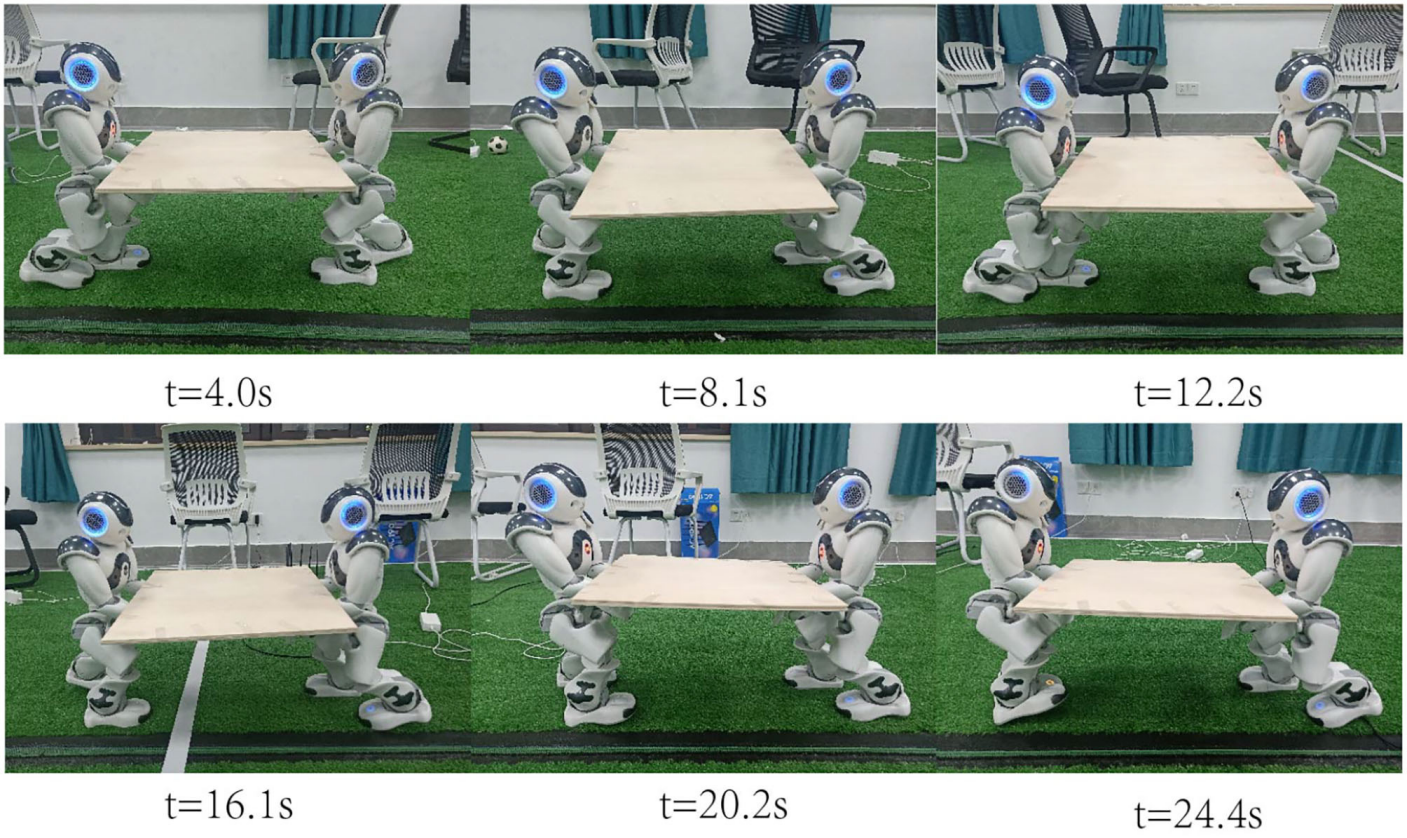
Humanoid robots cooperatively carry a heavy object.

**Figure 3:**
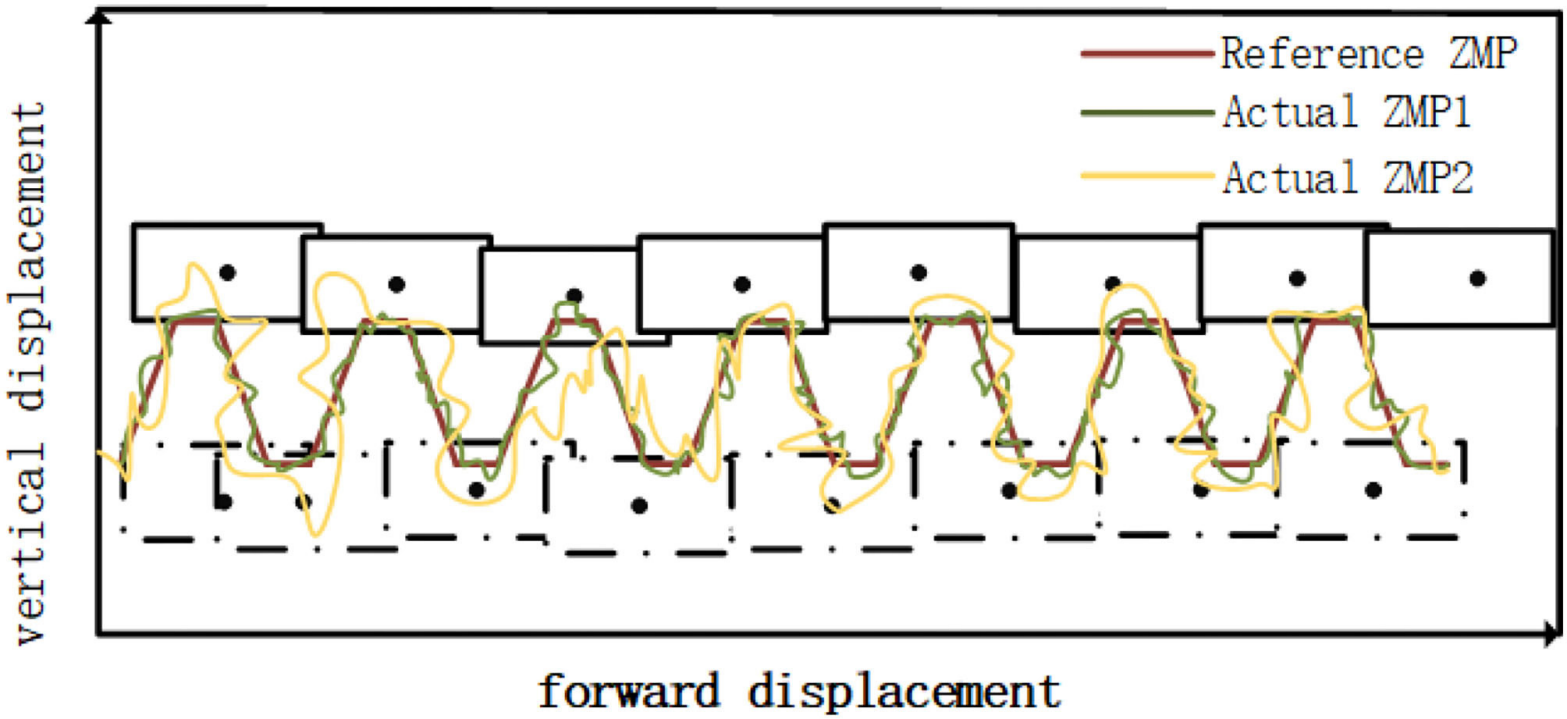
Comparison of ZMP trajectories during actual cooperative handling.

Based on the energy consumption index function, the optimal energy consumption solution that satisfies the robot cooperation in the stable area is searched. The 20 s process of robots' cooperative handling is shown in Figure 2, where each picture represents the pose at the given moment. Taking the forward posture of the robot on the left as an example, the reference ZMP trajectory of its cooperative handling and the ZMP trajectory under the two control methods are shown in Figure 3. The upper part of the lateral displacement is the landing location of the left foot, the black solid point is the landing location, Reference ZMP denotes reference ZMP trajectory, Actual ZMP1 denotes ZMP trajectory based on the parameterized control, and Actual ZMP2 denotes ZMP trajectory under normal control. It can be seen from Figure 3, that the jitter phenomenon is observed in the actual ZMP trajectory regardless of the control method. However, the swing amplitude of the ZMP trajectory under the parameterized control and the uncertainty factor are both less significant. In addition, the stability is found to be better based on the ZMP trajectory. The ZMP trajectory under the parameterized control follows the reference trajectory well, thus ensuring stronger stability of the robot collaboration process.

For the collaborative movement of humanoid robots, time consumption is an important indicator to evaluate its performance. The shorter the time consumed, the higher the collaboration efficiency achieved. By comparing the time consumed by the normal control method and the parameterized control method as follows:

Table 2 shows the consumption time and average time of the two control methods in 1, 5, 10, 15, and 20 walking cycles. After completing 20 walking cycles, the average unit cycle time of the normal control is 3.81 s, and the average unit cycle time of the parameterized control is 4.19 s, respectively. The average unit time difference between the two control methods is 0.38 s, while the total time difference is 7.6 s. In general, a small amount of time difference has insignificant effect on the performance of the collaboration. However, it is reasonable to compromise the amount of time to achieve higher stability. As a result, the parameterized control is feasible for the collaboration of humanoid robots.

**Table 2:** The unit cycle consumption time of the parameterized control and normal control.

**Number of cycles**	**Normal control**		**Parameterized optimization control**	
	**Time consumed/s**	**Average unit time /s**	**Time consumed /s**	**Average unit time /s**
1	3.7	3.70	4.1	4.10
5	19.3	3.86	20.9	4.18
10	38.0	3.80	41.8	4.18
15	58.0	3.87	62.4	4.16
20	76.1	3.81	83.7	4.19

### 5.2. Optimal Control Analysis

**Figure 4:**
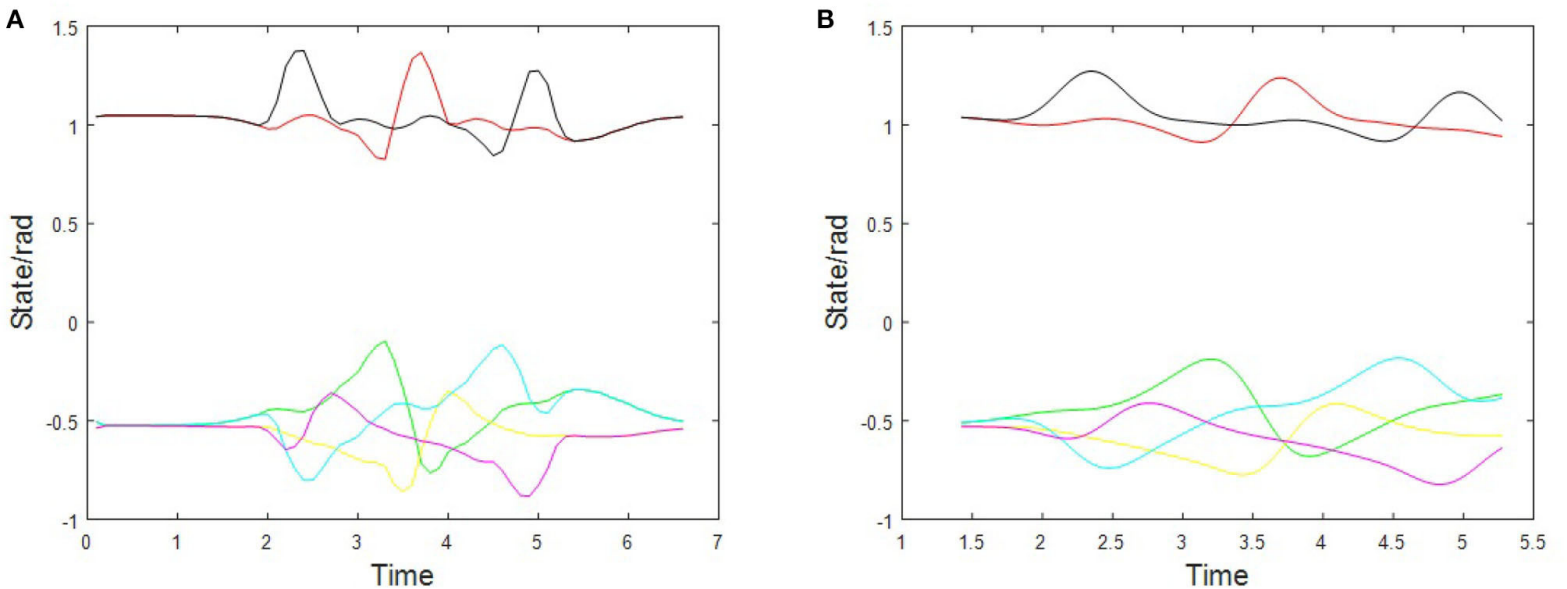
The time series curve of each state variable. **(A)** The normal control, **(B)** the parameterized control.

Figure 4 illustrates the time series curve of each joint simulated by MATLAB. Figure 4A shows the hip joint of the left leg (green), the hip joint of the right leg (bright cyan), the knee joint of the left leg (red), the knee joint of the right leg (black), the ankle joint of the left leg (yellow), and the ankle joint of the right leg (manganese purple).

In the time curve of state variables without optimal control, Figure 4B is the state curve of each control variable based on the optimal control method during the time in the black box of Figure 4A. It can be seen from the figure that after adopting the parameterized control method, the slope of the time curve of each state variable is smaller and smoother without sudden changes. However, the normal control method has undergone major changes, which will cause joint mutations and make the robot lose stability during walking. Therefore, the optimal control is realized.

### 5.3. Energy Consumption Analysis

Table 3 shows the energy consumption per unit cycle of the hip, knee and ankle joints of the NAO robot walking in a forward posture under normal control and parameterized optimization control, respectively. According to the presented results, the energy consumption per unit cycle of each joint after the application of the parameterized optimization is 17–22%, which is lower than that of the normal control. For the total energy consumption of all joints in a unit cycle, the parameterized control is about 20%, which is lower than the normal control. It is hence proved that the parameterized control can effectively reduce energy consumption. Due to the application of the parameterized control, high energy consumption caused by sudden changes in joints is avoided, resulting in more stable movements of the joints to consume less energy. Therefore, it is effective to use the parameterized control to reduce energy consumption under cooperative tasks.

**Table 3:** Energy consumption per unit cycle of each joint under two types of controls in the actual cooperative handling process.

**Joint**	**Normal control**	**Parameterized optimization control**	**Reduction/%**
	**Energy expenditure/J**		
Left hip	31.1	25.4	18.33
Right hip	33.8	26.3	22.19
Left knee	27.9	22.5	19.35
Right knee	26.6	21.9	17.67
Left ankle	25.1	19.8	21.12
Right ankle	25.9	20.1	22.39
Total energy	170.4	136.0	20.19

**Figure 5:**
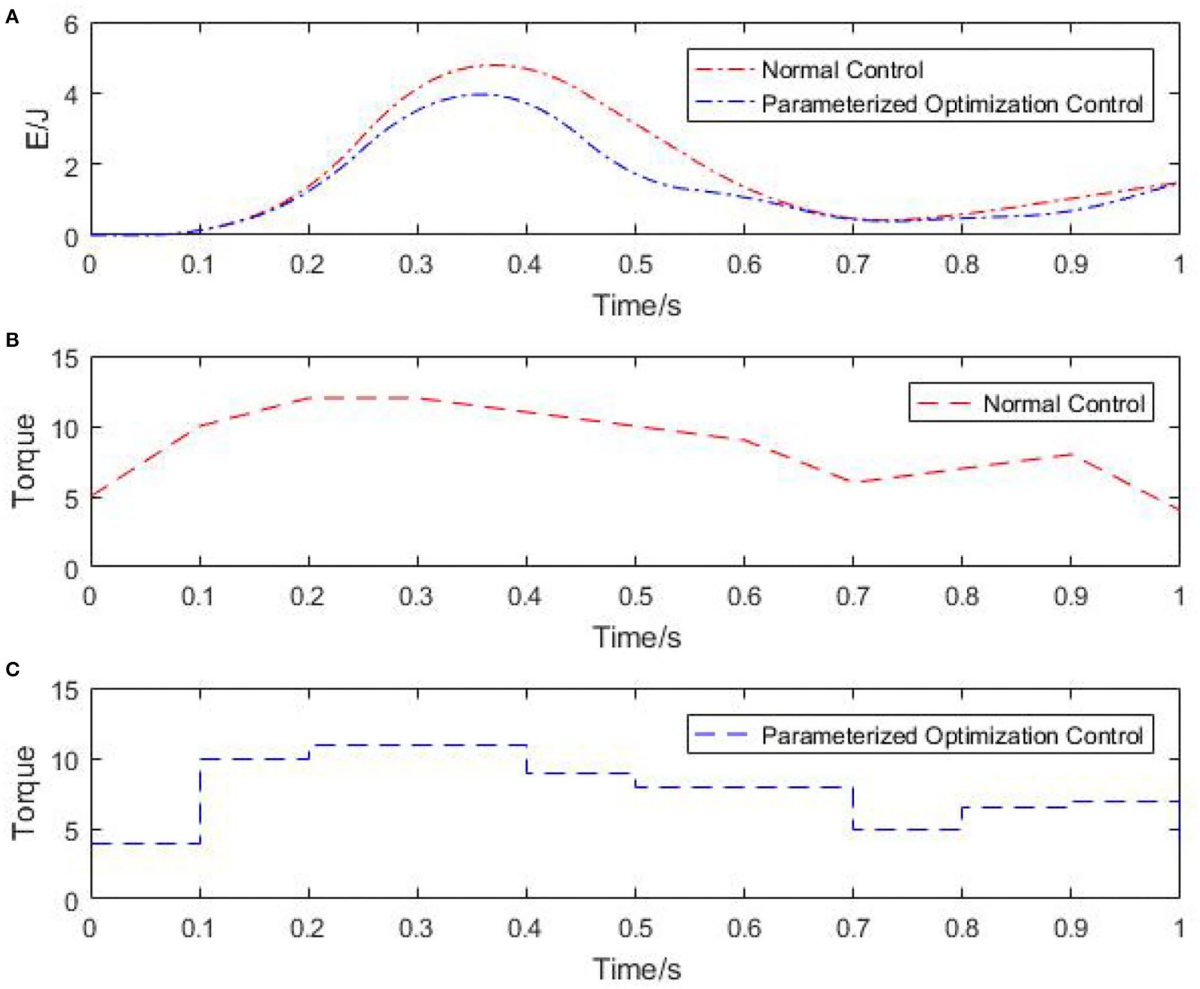
Comparison of energy consumption and control torque under the two control methods. **(A)** Energy consumption under two control methods. **(B)** Control torque under the normal control. **(C)** Control torque under parameterized optimization control.

Figure 5 shows the energy consumption assessment diagram of the two robots under different control methods and the change diagram of the output torque over time. It can be seen that the energy consumption change trend is basically the same under different control methods in Figure 5A. But compared with normal control in Figure 5B, the energy consumption of parameterized optimization control in Figure 5C is smaller and lower. Therefore, it is effective to use the parameterized control to reduce energy consumption.

## 6. Conclusion

In this paper, the optimal parameterized control method is used to optimize the energy consumption in the cooperative movement of humanoid robots under the premise of satisfying the stability judgment index of the robot during the movement. Experimental findings show that the humanoid robot can always maintain stability during the collaboration process. And from the optimal control of each joint and the analysis of energy consumption per unit period, the energy expenditure of the parametric control is reduced by 20% compared with the normal control. Although, the unit cycle consumption time of the parameterized control method is slightly more than the normal control method, the energy consumption of the parameterized control method is smaller and the joint trajectory tends to be smooth, which can more ensure the stability of the robot cooperation. It demonstrates that the method proposed in this paper is efficient. In future work, the proposed parameterized control method would be further modified and applied to the stability control of the arm joints of the humanoid robot in cooperative tasks.

## Data Availability Statement

The raw data supporting the conclusions of this article will be made available by the authors, without undue reservation.

## Author Contributions

QZ and YL conducted the experiment, designed the study, and analyzed the data. QZ, CZ, and TS wrote the manuscript. All authors contributed to the article and approved the submitted version.

## Conflict of Interest

The authors declare that the research was conducted in the absence of any commercial or financial relationships that could be construed as a potential conflict of interest.

## Footnotes

^1^https://developer.softbankrobotics.com/nao6/nao-documentation
